# Cervical Thymic Cyst in an Adult

**DOI:** 10.1155/2014/801745

**Published:** 2014-05-04

**Authors:** Hassan A. Alzahrani, Javeria M. Iqbal, Amani K. Abu Shaheen, Bandar N. Al Harthi

**Affiliations:** Department of Surgery, King Fahad Medical City, P.O. Box 59046, Riyadh 11525, Saudi Arabia

## Abstract

Cervical thymic cysts (CTCs) are unusual lesions, representing only 1% of cystic cervical masses. Diagnosis of this condition in adults is even rarer. We report a 34-year-old female who presented with asymptomatic progressively growing left-sided neck swelling. Neck ultrasound (US) showed a large cystic lesion with septation, compressing the ipsilateral vessels. Magnetic resonance imaging (MRI) confirmed the US findings. Surgical excision was performed which subsequently showed findings consistent with CTC. CTC in adult is extremely rare, with few reported cases identified in the literature. Thymic gland anomalies in the neck are the consequences of an arrest in the descent of the gland, sequestration of the thymic tissue, or failure of involution. The diagnosis of this condition is rarely done prior to surgical excision. The clinical presentation, radiologic imaging, surgical findings, and histologic appearance are all essential components to make the correct diagnosis of this very rare differential diagnosis of cystic lateral neck swelling.

## 1. Background

Cervical thymic cysts (CTCs) are unusual lesions usually presenting in the 1st decade of life and representing only 1% of cystic cervical masses [1]. Most patients with thymic cysts complain of a slowly enlarging asymptomatic cervical mass with few reported cases in adults [2].

We report here a rare case of a patient with CTC in an adult that was diagnosed after being excised, describing radiological, intraoperative, and histopathological findings. Moreover, CTC is seldom considered in the differential diagnosis of the neck masses preoperatively, making it an interesting case for publication.

## 2. Case Report

**Figure 1:**
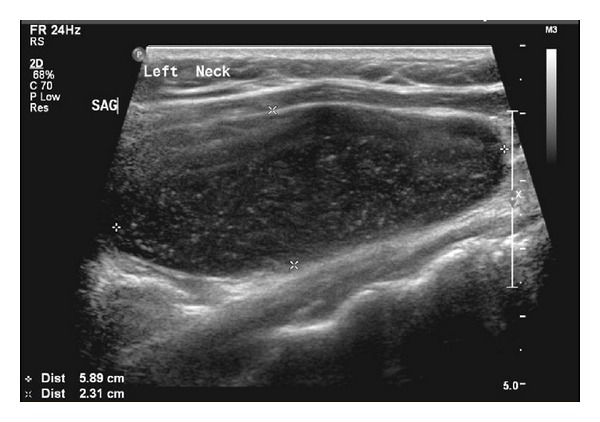
Ultrasound-sagittal view showing the large cystic lesion.

**Figure 2:**
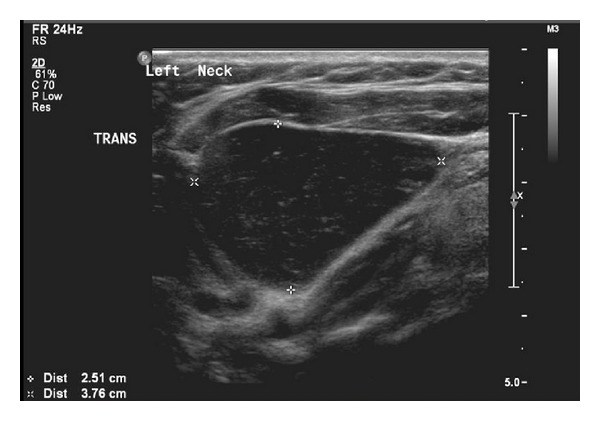
Ultrasound-transverse view showing the large cystic lesion.

**Figure 3:**
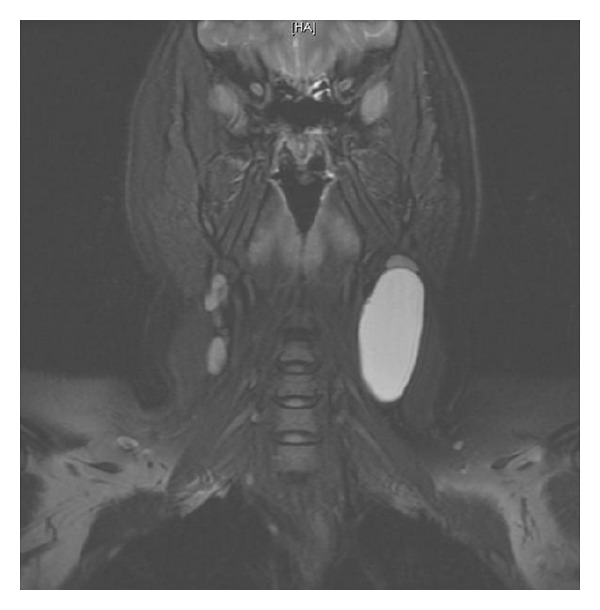
MRI-coronal view showing the cystic lesion with high T2 signal.

**Figure 4:**
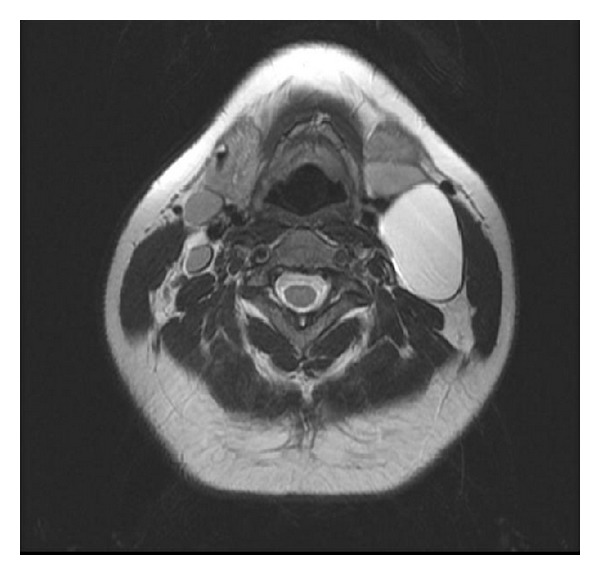
MRI-axial view showing the cystic lesion with high T2 signal.

Our case was a 34-year-old female not known to have any chronic medical illnesses. The patient was referred to our hospital from the primary care center for left-sided neck swelling of 1-year duration. The swelling was discovered incidentally and gradually increased in size over time with no history of pain, increased body temperature, discharges, loss of appetite, change of voice, or pressure symptoms. There was no significant family history. Physical examination revealed a left-sided neck swelling, 6 × 3 cm, nontender, oval in shape, firm in consistency, and not pulsatile or increased with Valsalva maneuver and was mostly located beneath the sternocleidomastoid muscle. On laboratory workup, she was not found to have any related abnormalities. Neck US showed a large cystic lesion with septation measuring 5.9 × 2.3 cm, compressing the ipsilateral vessels, with no vascularity (Figures 1 and 2). Neck MRI showed findings highly consistent with branchial cleft cyst with reactive lymph nodes (Figures 3 and 4).

**Figure 5:**
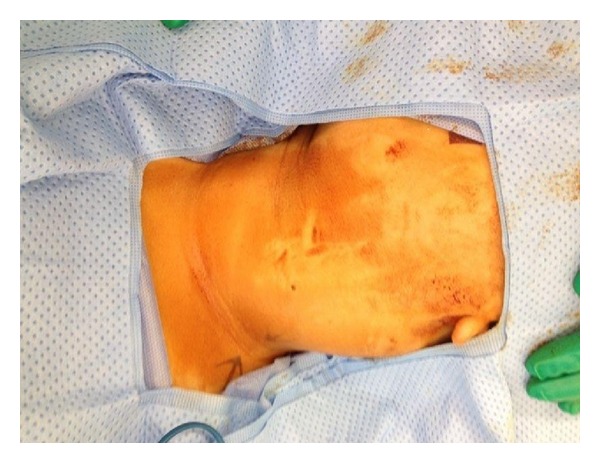
Intraoperative picture showing the mass before starting the skin incision.

**Figure 6:**
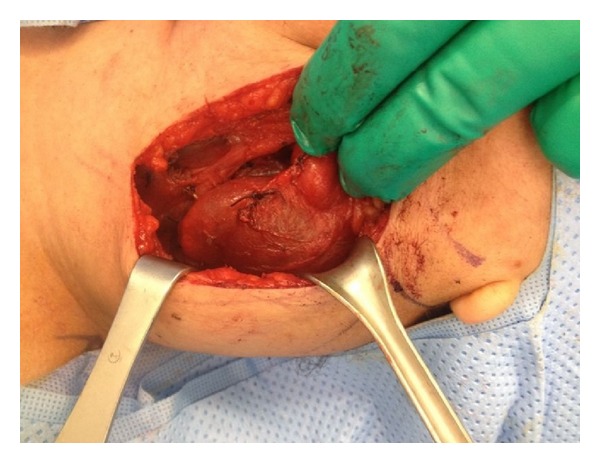
Intraoperative picture showing the lesion underneath the sternocleidomastoid muscle compressing medially on left internal jugular vein.

**Figure 7:**
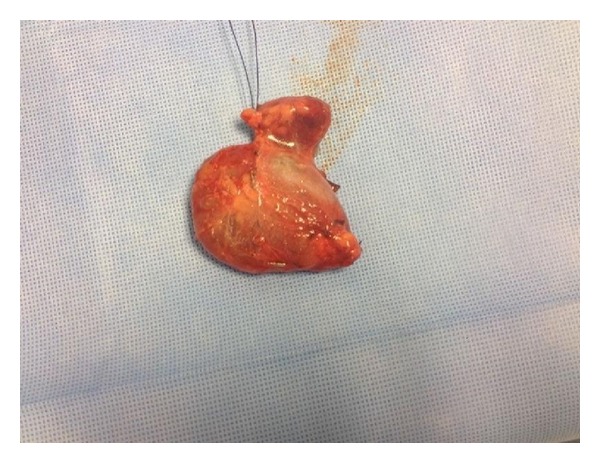
Intraoperative picture showing the cyst after removal with leaking clear fluid content.

The patient was admitted and scheduled for surgery on day 2 of admission. Surgical approach was through a vertical incision medial to sternocleidomastoid muscle whereas a large cyst has been found adherent to the carotid sheath medially and to the sternocleidomastoid muscle anterolaterally (Figures 5, 6, and 7). The complete excision was successfully and safely achieved. Histopathological examination of the mass showed a cyst mass with a gray tan smooth outer surface. On opening, it was unilocular cyst filled with clear fluid and lined with partially pale and partially brown, slightly nodular lining. Microscopical examination showed a cyst lined with attenuated squamous epithelium and showed lymphoid tissue in the wall, with adjacent thymic tissue and reactive lymph node.

The postoperative period was uneventful with no complications, and the patient was discharged on day 2 postoperatively.

## 3. Discussion

Thymus gland is mainly originated from the third pharyngeal pouch in association with the inferior parathyroid glands. It is the main organ of the lymphoid system during infancy. Right and left portions of the thymic primordium descend down the neck during the sixth to eighth weeks of gestational life and fuse to form the gland [3]. Finally, thymus gland reaches its destination in anterior mediastinum behind the sternum. Thymic gland anomalies in the neck are the consequences of an arrest in the descent of the gland, sequestration of the thymic tissue, or failure of involution [4]. As thymus gets atrophied after puberty, about two-thirds of these lesions occur during the first decade of life [5]. They can be seen at any level of normal thymic descent from the mandible to the mediastinum. Fifty percent of these cystic masses are continuous with mediastinal thymus [6]. CTCs are more frequent in males, usually asymptomatic and left-sided, and are located anteriorly and deep to the middle third of the sternocleidomastoid muscle [7].

There were five theories descried for the development of CTCs as follows[8]: (1) remnants of branchial clefts or the thymopharyngeal tract, (2) sequestration of thymic tissue during migration, (3) neoplastic change in the lymphoid or surrounding tissues, (4) cystic degeneration of Hassall's corpuscles, and (5) lymphoid tissue that has arrested in various stages of thymic development. Nowadays, the most believed etiologies of the CTCs are either congenital, due to persistence of the thymopharyngeal duct, or acquired, due to degeneration of Hassall's corpuscles within the remnants of ectopic thymus [9, 10].

The diagnosis of cervical thymic tissue is rarely made preoperatively [2]. The differential diagnosis of a cervical mass includes thyroglossal duct cyst, brachial cleft cyst, cervical lymphadenopathy, benign tumors (dermoid, epidermoid, hemangioma, and lymphangioma), and malignant tumors (lymphoproliferative, soft tissue sarcomas and other metastatic lesions) [11].

Three major imaging modalities are used to aid diagnosis. Ultrasound has been reported as useful in distinguishing cystic versus solid structures, assessing proximity to the carotid sheath, and, in some instances, identifying thymic tissue specifically by echo patterns [12]. CT gives important information to distinguish thymic cysts from other congenital anomalies such as lymphangiomas and branchial cleft cysts based on specific anatomic location and appearance. CT also provides information regarding proximity to vital structures that optimizes operative planning. A contrast study shows a homogeneous hypodense mass with minimal rim enhancement when imaging congenital thymic cysts [13]. The MRI shows low T1 signal and high T2 signal as well as superior soft tissue definition. This study is particularly useful in imaging a fistula tract connecting to the pharynx as well as determining the relationship between the cyst and mediastinal thymus [14].

Surgery is the treatment of choice and helps to establish the definitive diagnosis [2]. Transverse cervical neck incision is the most common approach, although vertical approaches paralleling the anterior sternocleidomastoid border have been reported [15]. The excision of the mass should be performed with great care because of the close anatomical relationships of CTCs with large vessels and neck nerves (carotid artery, jugular vein, vagus nerve, glossopharyngeal nerve, hypoglossal nerve, phrenic nerve, and recurrent laryngeal nerve); particularly, if there is adherence of the CTC with those structures, CTCs may also present surgical challenges if the cyst extends into the mediastinum. In this situation, a median sternotomy may be required to remove the entire specimen [16].

Histopathologically, thymic cysts are unilocular or multilocular containing brownish fluid. The cyst wall lining ranges from flattened squamous or cuboidal cells to multilayered stratified squamous epithelium and to even primitive respiratory epithelium. Lobulated lymphoid tissue in the cyst wall contains Hassall's corpuscles [17].

## 4. Conclusion

CTC in adults is extremely rare, with few reported cases identified in the literature. The clinical presentation, radiologic imaging, surgical findings, and histologic appearance are all essential components to make the correct diagnosis of this very rare differential diagnosis of lateral cystic neck swelling.

## References

[1] Bruyère PJ, Moreau P, Ghaye B (2007). Congenital thymic cyst. *Journal Belge de Radiologie*.

[2] Michalopoulos N, Papavramidis TS, Karayannopoulou G (2011). Cervical thymic cysts in adults. *Thyroid*.

[3] Sturm-O’Brien AK, Salazar JD, Byrd RH (2009). Cervical thymic anomalies: the Texas children’s hospital experience. *Laryngoscope*.

[4] Tovi F, Mares AJ (1978). The aberrant cervical thymus. Embryology, pathology, and clinical implications. *American Journal of Surgery*.

[5] Terzakis G, Louverdis D, Vlachou S, Anastasopoulos G, Dokianakis G, Tsikou-Papafragou A (2000). Ectopic thymic cyst in the neck. *Journal of Laryngology and Otology*.

[6] Saggese D, Ceroni Compadretti G, Cartaroni C (2002). Cervical ectopic thymus: a case report and review of the literature. *International Journal of Pediatric Otorhinolaryngology*.

[7] Millman B, Pransky S, Castillo J, Zipfel TE, Wood WE (1999). Cervical thymic anomalies. *International Journal of Pediatric Otorhinolaryngology*.

[8] Speer FD (1938). Thymic cysts. *Bulletin of the New York Medical College*.

[9] Berenos-Riley L, Manni JJ, Coronel C, De Wilde PCM (2005). Thymic cyst in the neck. *Acta Oto-Laryngologica*.

[10] Cigliano B, Baltogiannis N, De Marco M (2007). Cervical thymic cysts. *Pediatric Surgery International*.

[11] Kacker A, April M, Markentel CB, Breuer F (1999). Ectopic thymus presenting as a solid submandibular neck mass in an infant: case report and review of literature. *International Journal of Pediatric Otorhinolaryngology*.

[12] Han BK, Yoon H-K, Suh Y-L (2001). Thymic ultrasound. II. Diagnosis of aberrant cervical thymus. *Pediatric Radiology*.

[13] Choi YW, McAdams HP, Jeon SC (2001). Idiopathic multilocular thymic cyst: CT features with clinical and histopathologic correlation. *American Journal of Roentgenology*.

[14] Molina PL, Siegel MJ, Glazer HG (1990). Thymic masses on MR imaging. *American Journal of Roentgenology*.

[15] Vade A, Griffiths A, Hotaling A, Eisenbeis JF, Husain AN (1994). Thymopharyngeal duct cyst: MR imaging of a third branchial arch anomaly in a neonate. *Journal of Magnetic Resonance Imaging*.

[16] Ridder GJ, Boedeker CC, Kersten AC (2003). Multilocular cervical thymic cysts in adults. A report of two cases and review of the literature. *European Archives of Oto-Rhino-Laryngology*.

[17] Koeller KK, Alamo L, Adair CF, Smirniotopoulos JG (1999). From the archives of the AFIP. Congenital cystic masses of the neck: radiologic-pathologic correlation. *Radiographics*.

